# Integrated transcriptomic and proteomic analysis reveals the regulatory role of exogenous gibberellin in sugarcane internode maturation

**DOI:** 10.3389/fpls.2026.1831439

**Published:** 2026-06-05

**Authors:** Rongfa Chen, Zongmeng Wu, Zhenguang Lai, Junli Wen, Weiqing Dong, Jie Liao, Yanyong Wu, Junyang Chen, Youxiong Que, Jianming Wu, Yegeng Fan

**Affiliations:** 1 Sugarcane Research Institute, Guangxi Academy of Agricultural Sciences/Guangxi Key Laboratory of Sugarcane Biotechnology and Genetic Improvement, Ministry of Agriculture and Rural Affairs/Guangxi Key Laboratory of Sugarcane Genetic Improvement/State Key Laboratory for Conservation and Utilization of Subtropical Agro-bioresources, Nanning, China; 2 Guangxi Ecological Engineering Vocational College, Liuzhou, China; 3 Tropical Biotechnology Institute of Chinese Academy of Tropical Agricultural Sciences, Haikou, China

**Keywords:** integrated analysis, phenylalanine ammonia-lyase, phenylalanine metabolism, phenylpropanoid biosynthesis, sugarcane internode

## Abstract

**Introduction:**

Sugarcane cultivation is a vital component of the agricultural economy in southern China. Investigating internode development in sugarcane is crucial for optimizing cultivation management practices and improving cane yield.

**Methods:**

In this study, transcriptome and proteome sequencing were performed on internode tissues of sugarcane cultivar Guitang 42 at 0, 6, and 12 days post-treatment, aiming to identify key molecular components and elucidate biological pathways through which exogenous gibberellic acid (GA_3_) regulates internode maturation.

**Results:**

Accordingly, GA_3_ predominantly promoted internodal elongation, rather than nodal expansion. Following transcriptome and proteome sequencing, 3D principal component analysis (PCA) based on both datasets revealed a clear separation between the GA_3_-treated (GA) and control (CK) groups. The comparison of GA_6d vs. CK_0d identified the largest number of differentially expressed genes (DEGs, 34,541), followed by CK_12d vs. CK_0d (27,898) and GA_12d vs. CK_0d (22,709). Similarly, the pairwise comparison between GA_6d and CK_0d yielded the highest number of differentially expressed proteins (DEPs, 363). KEGG enrichment analysis based on DEGs, DEPs and their intersection revealed that GA_3_ treatment up-regulated the phenylpropanoid biosynthesis and phenylalanine metabolism pathways, thereby promoting lignin biosynthesis. Additionally, PPI analysis revealed high-confidence interactions between two hub proteins (PAL and 4CL). Finally, we elucidated the biosynthetic pathways that produce p-hydroxyphenyl lignin, guaiacyl lignin, and syringyl lignin using L-phenylalanine as the substrate.

**Discussion:**

The results presented herein provide new insights into sugarcane internode maturation.

## 1 Introduction

Sugarcane, a member of the genus *Saccharum*, is a primary feedstock that contributes over 80% of the sugar output and 40% of the bioethanol production worldwide. The principal sugarcane cultivation regions in China are geographically situated between latitudes 18.5° N and 32° N and longitudes 92° E and 122° E, encompassing major provinces and autonomous regions, including Guangxi, Yunnan, Guangdong, and Hainan (Luo et al., 2015). Statistical data spanning the period from 1961 to 2013 demonstrates a remarkable expansion of China’s sugarcane industry. During these years, the national sugarcane yield surged from 2.643 million metric tons to 126.13 million metric tons; the total cultivation area expanded substantially from 108,000 hectares to 1.827 million hectares; the per-hectare production rose from 24.0 metric tons to 67.4 metric tons; and the aggregate sugar output increased dramatically from 0.15 million metric tons to 10.613 million metric tons (Zhang and Govindaraju, 2018).

A sugarcane stalk is structurally composed of multiple differentiated internodes and nodes arranged alternately in an orderly manner. Following the tillering stage, characterized by the sprouting of new shoots from the base of the parental stalk, sugarcane undergoes a phase of vigorous vegetative growth with prominent stalk elongation (Lingle and Thomson, 2012); cell division and internodal cell proliferation drive the radial and longitudinal expansion of the stalk. In contrast, during the maturation stage, cell elongation decelerates substantially, and the cell walls of parenchyma cells are progressively deposited with cellulose, hemicellulose, and lignin (Kim and Day, 2011), while sucrose accumulation is initiated intracellularly (Pereira et al., 2017). Internode elongation and maturation directly determine the biomass and sucrose yield in sugarcane.

Plant growth regulators (PGRs) are a class of synthetic compounds capable of modulating plant growth, production, and quality by regulating processes such as seed germination, stem and hypocotyl elongation, leaf morphogenesis, floral development, and fruit ripening at low concentrations (McMillan et al., 2020). Some of them even mediate responses to biotic and abiotic stresses. The established gibberellin (GA) signaling mechanism involves the formation of a stable GA-GID1-DELLA complex and rapid degradation of DELLAs (Li et al., 2016). This degradation cascade relieves DELLA-mediated growth repression, thereby regulating cell division, cell elongation, and expression of related genes, ultimately modulating plant growth and yield. To date, research on the mechanisms underlying GA-mediated internode development in sugarcane has been predominantly based on transcriptomic approaches. Chen et al. demonstrated that metabolic processes, photosynthesis, and plant hormone signal transduction pathways are linked to internode elongation, while the cellulase synthase A9 (*CESA9*) gene contributes to cell wall biosynthesis (Chen et al., 2020). The label-free shotgun proteomics study by Boaretto et al. revealed that phenylalanine ammonia lyase (PAL) and 4 coumarate-CoA ligase (4CL) contribute to the development of sugarcane internodes by regulating lignin biosynthesis (Boaretto et al., 2021). However, exclusive reliance on transcriptomic analysis to delineate the biological insights underlying sugarcane internode development will inevitably overlook the interaction patterns and regulatory mechanisms among molecular components, which may further mislead breeders in field cultivation management, resulting in resource wastage.

Given this, the present study was designed focusing on the regulatory role of gibberellins in stalk elongation and internode development in plants. Guitang 42, a commercial sugarcane cultivar, was subjected to exogenous treatment with gibberellic acid (GA_3_). Before screening differentially expressed genes (DEGs) and differentially expressed proteins (DEPs), plant samples were collected at different time points for transcriptome and proteome sequencing. This study aimed to elucidate the multi-omics regulatory network underlying internode elongation and maturation in sugarcane, identify core regulatory genes and key functional proteins involved in GA signaling response and metabolic pathways, and clarify their expression patterns and interactive relationships during sugarcane internode development. This integrated multi-omics analysis circumvents the inherent limitations of single-omics approaches, unravelling the insights that remain concealed when individual omics datasets are analyzed in isolation (Sanches et al., 2024). Furthermore, it establishes an upstream-downstream link spanning from transcriptional regulation to protein functionality. The results presented here are expected to provide systematic insights into sugarcane internode maturation and offer further guidance for breeding practices.

## 2 Materials and methods

### 2.1 Plant materials and experimental design

The sugarcane cultivar Guitang 42, bred by the Sugarcane Research Institute (SRI) of Guangxi Academy of Agricultural Sciences, Nanning, China, was used in this study. Stalks from 10-month-old plants were collected, and the middle internodes were cut into single-bud set. The setts were immersed in warm water at 52 °C for 30 min to eliminate pathogens, then planted in moist sand boxes and placed in a climate-controlled chamber (Essenscien, USA). The growth conditions were set as follows: temperature 28.0 ± 0.1 °C, relative humidity 75 ± 1.5 %, photoperiod 12 h light/12 h dark (100 % full light, light intensity 25,000 lx). After emergence of the first two leaves, the seedlings were transplanted into plastic pots (35 cm × 35 cm × 50 cm, width × length × height), with two seedlings per pot. The potting mixture consisted of garden soil: leaf mould (or peat moss): coarse river sand (or perlite) at a ratio of 4: 3: 3 (v/v/v). At planting, 20 g of balanced compound fertilizer (N: P_2_O_5_: K_2_O = 15: 15: 15) was added per pot. At the six-leaf stage, an additional 25 g of the same fertilizer was top-dressed per pot. The volumetric soil water content was maintained between 18 % and 28 %. Plants were cultivated in a greenhouse at the Guangxi Key Laboratory of Sugarcane Genetic Improvement, Guangxi Academy of Agricultural Sciences, following established management practices. When plants had developed 9–10 leaves uniform plants were selected for the experiment and the internode tissue enclosed by the second leaf from the apex (the +2 internode) was marked on each plant.

At this stage, two treatments were established: foliar spray with water (control, CK) and foliar spray with 200 mg/L GA_3_ (Qiu et al., 2019; Chen et al., 2020). Based on preliminary time-course experiments, GA_3_-induced internode elongation was most dynamic at 6 days post-treatment (rapid response phase) and reached a plateau by 12 days (stabilization phase); therefore, these two time points were selected for sampling. Prior to spraying, the marked internode tissue was sampled from a separate batch of three plants (0 d). Immediately after sampling, the remaining plants were sprayed with either water or the GA_3_ solution. At 6 and 12 days post-treatment, internode tissue was collected from six independent plants per treatment and time point. The +2 internode was selected as the focal sampling site because it corresponds to the active elongation phase that has not yet entered terminal lignification, providing high sensitivity to GA_3_-induced developmental shifts, as was used in previous sugarcane GA_3_ studies (Wu et al., 2009; Chen et al., 2020). Tissue from three of these plants was processed as three independent biological replicates, with the remaining three plants kept as biological backup. Samples were flash-frozen in liquid nitrogen and stored at -80 °C for subsequent transcriptomic and proteomic analyses. The stalk diameter and internode length were recorded at each sampling time point. The experimental groups were designated as groups CK_0d, CK_6d, GA_6d, CK_12d, and GA_12d, respectively.

### 2.2 Transcriptome sequencing and analysis

Total RNA was extracted from internode tissues using TRIzol^®^ Reagent (Magen). The RNA concentration and purity were assessed using a Nanodrop ND-2000 spectrophotometer (Thermo Scientific, USA), and RNA integrity was evaluated using an Agilent Bioanalyzer 4150 (Agilent Technologies, CA, USA). Following quality control, PE libraries were constructed using the ABclonal mRNA-seq Lib Prep Kit (ABclonal, China), according to the manufacturer’s instructions. Briefly, mRNA was enriched from 1 µg of total RNA using oligo(dT) magnetic beads and fragmented in first strand synthesis reaction buffer. First-strand cDNA was synthesized using random primers and reverse transcriptase (RNase H), followed by second-strand synthesis using DNA Polymerase I, RNase H, and dNTPs. The double-stranded cDNA fragments were ligated with adapters and amplified by PCR. The final libraries were purified and their quality was assessed on an Agilent Bioanalyzer 4150 (Agilent Technologies, CA, USA). Sequencing was performed on a MGISEQ-T7 platform (MGI Tech, Shenzhen, China) using PE150 reads.

Raw sequencing reads were quality-trimmed using Cutadapt (v4.0) (Martin, 2011). Clean reads were then aligned to the reference genome (Saccharum_hybrid R570, https://sugarcane.gxu.edu.cn/scdb/download, v2023) using HISAT2 (v2.2.1) (Kim et al., 2015), and gene expression was quantified with featureCounts from the Subread package (v2.0.8) (Liao et al., 2014) using the corresponding GFF3 annotation file (https://sugarcane.gxu.edu.cn/scdb/download, R570.v2023.gff3). Differential expression analysis was conducted using DESeq2 (v1.46.0) (Love et al., 2014), applying a threshold of padj < 0.05 and |log_2_(fold change)| > 1 to identify DEGs. Functional enrichment analysis of DEGs for Gene Ontology (GO) terms and KEGG pathways was performed using clusterProfiler (v4.14.0) (Wu et al., 2021).

### 2.3 Proteome sequencing and analysis

Frozen samples were pulverized in liquid nitrogen and proteins were precipitated using the TCA/acetone method (Wiśniewski et al., 2009). The pellets were lysed in SDT buffer (4% SDS, 100 mM Tris-HCl, pH 7.6), followed by sonication and heating at 95 °C for 15 min. After centrifugation at 14,000 × g for 40 min, the supernatant was collected and protein concentration was determined using the bicinchoninic acid (BCA) assay. Proteins (15 µg per sample) were separated on a 4-20% SDS-PAGE gel at 180 V for 45 min and visualized with Coomassie Blue R-250.

Gel bands were excised, reduced with 40 mM dithiothreitol (DTT) (37 °C, 1.5 h), alkylated with 20 mM iodoacetamide (room temperature, 30 min in darkness), and digested with trypsin (1:50 w/w, 37 °C, overnight). The peptides were desalted using C18 cartridges, concentrated by vacuum centrifugation, and reconstituted in 0.1% formic acid. Peptide concentration was estimated by measuring the absorbance at 280 nm, and indexed Retention Time (iRT) peptides were spiked for retention−time calibration. Liquid chromatography-tandem mass spectrometry (LC−MS/MS) analysis was performed on an Orbitrap™ Astral™ mass spectrometer (Thermo Fisher Scientific, Bremen, Germany) coupled to a Vanquish Neo UHPLC system (Thermo Fisher Scientific, Waltham, MA, USA) in data−independent acquisition (DIA) mode (Li et al., 2017; Stewart et al., 2023). MS1 scans were acquired at a resolution of 240,000 (380-980 m/z). For DIA, 299 isolation windows (2 m/z width) were used, with higher-energy C-trap dissociation (HCD) collision energy set to 25 eV.

DIA data were processed using DIA-NN (version 1.8.1) with the following parameters: trypsin as the enzyme, one maximum missed cleavage, carbamidomethylation of cysteine as a fixed modification, and oxidation of methionine and N−terminal acetylation as variable modifications. Protein identification was considered confident with 1% false discovery rate (FDR) threshold. Normalized to total peak intensity, the processed quantitative data were then imported into SIMCA−P (version 14.1, Umetrics, Umeå, Sweden) for multivariate analysis, including Pareto−scaled principal component analysis (PCA).

DEPs were screened with the criteria of |log_2_fold change (FC)| > log_2_(1.5) and a p−value < 0.05 (Student’s t−test). The resulting protein sets were functionally annotated using Blast2GO (BLASTP 2.8.0+) (Götz et al., 2008) for Gene Ontology (GO) terms (Ashburner et al., 2000) and KOBAS (version 3.0) for KEGG pathways (Kanehisa and Goto, 2000; Kanehisa et al., 2012). Enrichment analysis of GO terms and KEGG pathways was performed using Fisher’s exact test (Subramanian et al., 2005).

### 2.4 Integrative analysis of DEGs and DEPs

To identify genes and proteins with concordant expression changes, DEGs and DEPs from the same pairwise comparisons were intersected using custom R scripts. The overlapping lists were subjected to KEGG pathway enrichment analysis using Fisher’s exact test (Subramanian et al., 2005).

To predict functional interactions among the proteins encoded by the intersecting DEGs and DEPs, we performed a homology-based PPI analysis. Because the STRING database (https://string-db.org) does not currently include *Saccharum* species, the protein sequences of all detected sugarcane proteins were aligned against the *Arabidopsis thaliana* proteome using BLASTP (E-value ≤ 1e−5, identity ≥ 40%). The best hit for each sugarcane protein was retained to obtain the corresponding *Arabidopsis* gene identifier. For each pairwise comparison, the list of intersecting genes (common to both DEGs and DEPs) was submitted to the STRINGdb package (v2.14.0) using *Arabidopsis* identifiers (Altschul et al., 1997). A confidence score threshold of 0.4 was applied to retain high-confidence interactions. The resulting interaction networks were exported and visualized using Cytoscape (v3.10.0) (Majeed and Mukhtar, 2023).

### 2.5 Short time-series expression miner trend analysis

DEGs and DEPs were subjected to time-series trend analysis using STEM software. The data were normalized using the Min-Max normalization method, and the maximum number of model profiles was set to 8, with all other parameters kept as default. Trends were analyzed separately for two series: Series 1- CK_0d, CK_6d, CK_12d; and Series 2- CK_0d, GA_6d, GA_12d. Genes and proteins exhibiting distinct expression and protein abundance trends between the two series were identified as trend-divergents. Functional enrichment analysis of these trend-divergent genes and proteins for GO terms and KEGG pathways was performed using Fisher’s exact test. The intersection of the enriched pathways from the DEG and trend-divergent protein analyses was defined as the core differential pathway set.

### 2.6 Quantitative real-time PCR analysis

RT-qPCR was performed to independently validate the expression patterns of key phenylpropanoid biosynthesis genes. For each treatment group (CK and GA_3_) at each time point (0, 6, and 12 d), three biological replicates were analyzed. Total RNA, extracted as described previously, was reverse-transcribed into first-strand cDNA using the RTase III Primer Flexible All-in-One Mix (with dsDNase) (Yu gong Biotech, China), according to the manufacturer’s instructions. Quantitative PCR was performed using the QuantiNova SYBR Green PCR Kit (QIAGEN, Germany) on an ABI 7500 Real-Time PCR System (Applied Biosystems, Foster City, CA, USA). The reaction protocol consisted of an initial heat activation step at 95 °C for 2 min, followed by 40 cycles of denaturation at 95 °C for 5 s and a combined annealing and extension at 60 °C for 30 s. The *GAPDH* gene was used as an internal reference for normalization. Relative gene expression levels were calculated using the 2^-ΔΔCt^ method. All primer sequences used in this study are listed in **Supplementary Table 1**.

### 2.7 Lignin content measurement

Lignin content was determined using the acetyl bromide method with a commercial kit (NMW0811, Norminkoda, China). Sugarcane internode samples were dried at 60 °C to constant weight, ground into fine powder, and passed through a 60−mesh sieve. Approximately 3 mg of the dried powder was weighed into a screw−cap tube. The sample was washed sequentially with 600 μL of Reagent I (followed by centrifugation at 10,000×g for 5 min), 600 μL of distilled water, and 600 μL of acetone to remove interfering substances. After discarding the supernatant, 250 μL of Reagent II (acetyl bromide solution) was added carefully along the tube wall. The tube was tightly sealed, secured with an explosion−proof clip, and incubated in a 70 °C water bath for 30 min with gentle shaking every 10 min to facilitate acetylation. After cooling to room temperature, 250 μL of Reagent III was added, mixed thoroughly, and centrifuged at 8,000 rpm for 5 min. An aliquot (10 μL) of the supernatant was mixed with 490 μL of glacial acetic acid. Finally, 200 μL of the mixture was transferred to a 96−well UV plate, and the absorbance was measured at 280 nm using a microplate reader. A blank control (using reagents only) was processed in parallel. The lignin content (mg/g dry weight) was calculated according to the formula: Lignin (mg/g) = (ΔA × V1)/(ϵ × d × W × V/V2) = 2.141 × ΔA/W, where ΔA is the difference in absorbance between the sample and blank, ϵ is the extinction coefficient of lignin (23.35 mL/mg/cm), d is the path length (0.5 cm), V1 is the final detection volume (0.5 mL), V is the volume of supernatant used (0.01 mL), V2 is the acetylation reaction volume (0.5 mL), and W is the sample dry weight (g). Three biological replicates were measured per treatment group.

### 2.8 Internode compression tests

The mechanical properties of sugarcane internodes were evaluated using a universal testing machine (AGS-X, Shimadzu, Japan). Plants were sampled at 0 and 6 days post-treatment (d). At each time point, five biological replicates per treatment (control and GA_3_-treated) were collected. From each plant, the marked internode (the second leaf-enclosed internode at the time of marking, corresponding to +2 internode at 0 d and +3 internode at 6 d) was excised. A 1.5-cm-long segment was cut from the middle of the internode. For axial compression, the segment was placed with its cut ends facing the compression plates (load applied along the stem axis). For radial compression, the segment was placed with its lateral surface facing the plates (load applied perpendicular to the stem axis). A constant compression speed of 10 mm/min was applied until the maximum load was reached. The maximum load (N) and the corresponding displacement (mm) were recorded for each test, and compressive strength (MPa) was calculated as the maximum load (N) divided by the cross-sectional area (mm^2^) of the internode segment. The cross-sectional area was determined from the diameter measured at the mid-point of the segment, assuming a circular cross-section.

### 2.9 Statistical analysis

For phenotypic parameters (plant height, internode length, stalk diameter) and RT−qPCR data, comparisons between the GA_3_−treated and control groups at each time point were conducted using two−tailed Student’s t−test for independent samples. The compressive strength comparisons between the GA_3_−treated and control groups were performed using Student’s t-test (Levene’s test confirmed equal variances). The significance threshold was set at *p* < 0.05. For lignin content, one−way analysis of variance (ANOVA) followed by Tukey’s honestly significant difference (HSD) post−hoc test was performed to compare all five experimental groups (CK_0d, CK_6d, CK_12d, GA_6d, GA_12d). Differences were considered statistically significant at *p* < 0.05.

## 3 Results

### 3.1 Exogenous GA_3_ differentially regulates internode elongation and stalk diameter

**Figure 1:**
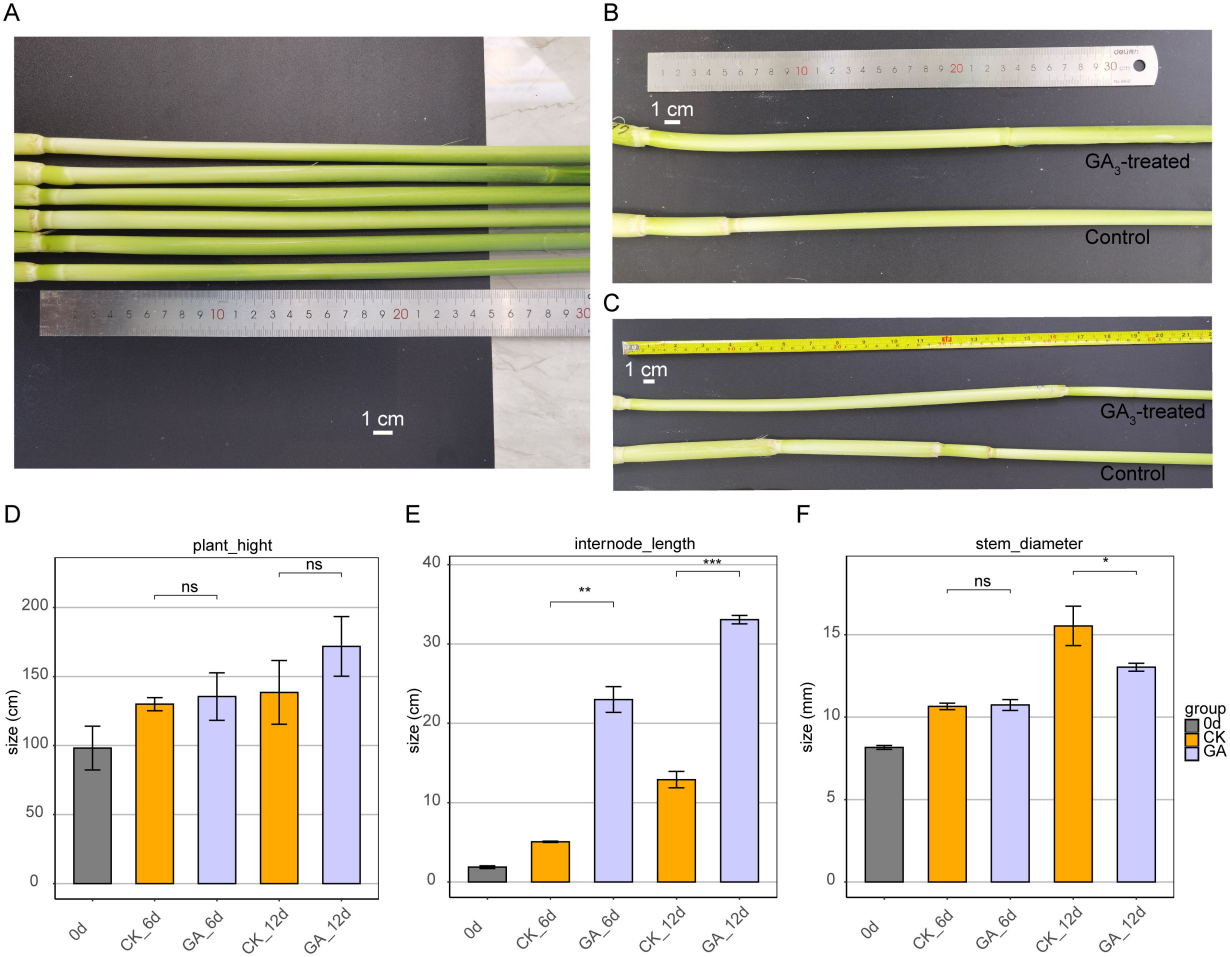
Phenotypic changes in sugarcane internodes in response to exogenous GA_3_ treatment. **(A-C)** Representative images of internodes from control and GA_3_-treated plants at day 0 **(A)**, day 6 **(B)**, and day 12 **(C)**. In panels B and C, the upper part shows GA_3_-treated internodes, and the lower part shows control internodes. **(D-F)** Quantitative analysis of plant height **(D)**, internode length **(E)**, and stalk diameter **(F)**. Data are presented as bar plots with error bars representing standard deviation (n=3). Significant differences between control and GA_3_-treated groups at each time point were determined by Student’s t-test (*p < 0.05, **p < 0.01, ***p < 0.001).

Phenotypic observations of plant height, internode length, and stalk diameter were conducted at 0, 6, and 12 days post-treatment (**Figures 1A–C**). At day 0, no significant differences were observed between the groups subsequently assigned to control and GA_3_ treatments for any of the measured parameters (**Figures 1A, D-F**). Following GA_3_ application, plant height showed no significant differences between treated and control groups at any time point (**Figure 1D**). However, internode length in the treated group was significantly greater than that in the control at both 6 and 12 days (**Figures 1B, E**; Student’s t-test, **p < 0.01, and ***p < 0.001). By the 12th day post-treatment, the stalk diameter in the treated group exhibited became significantly smaller compared to the control (**Figures 1C, F**; Student’s t-test, *p < 0.05).

### 3.2 Transcriptomic changes during GA_3_-induced internode maturation

**Figure 2:**
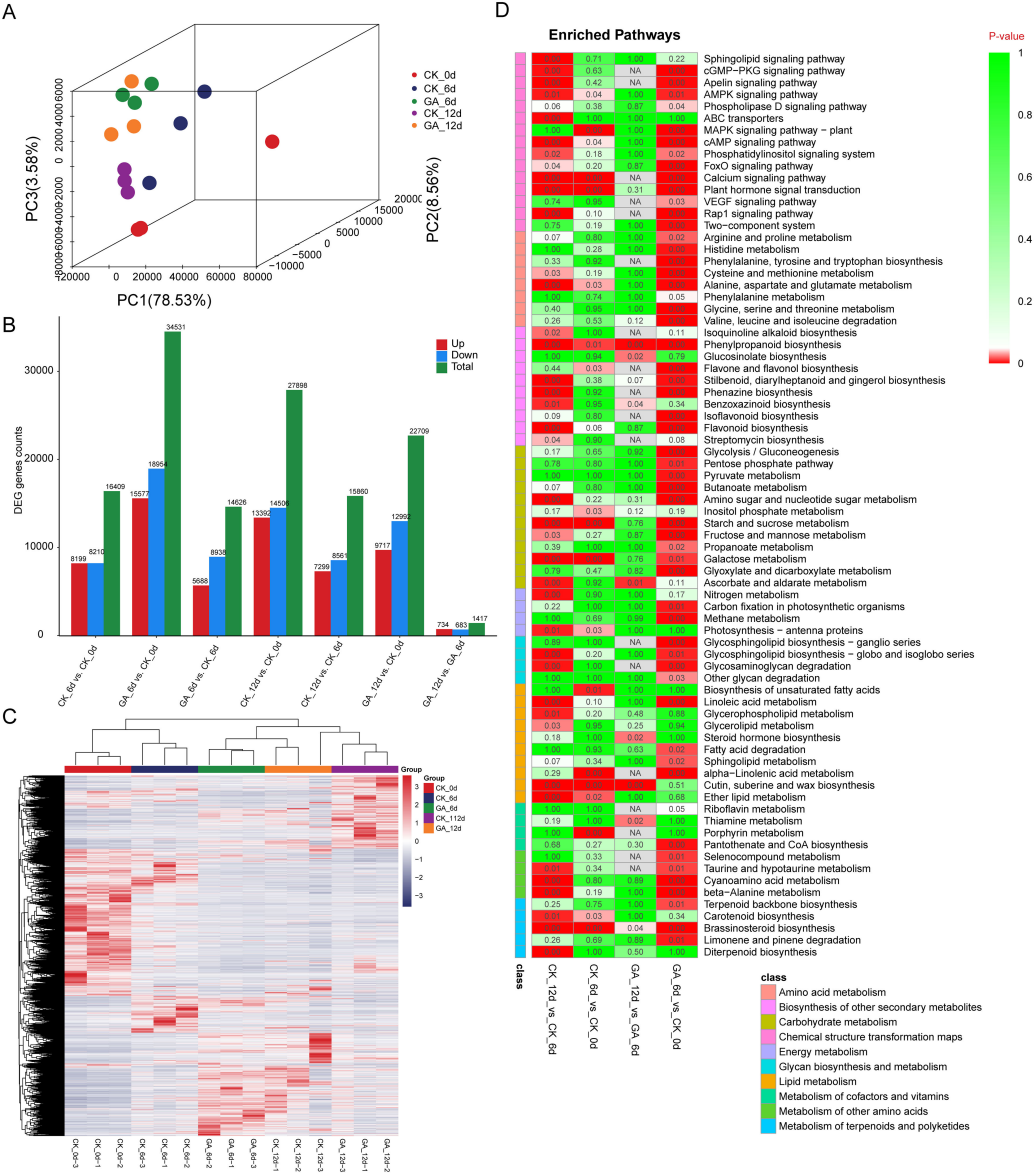
Transcriptomic profiling of sugarcane internodes during development and GA_3_ response. **(A)** 3D principal component analysis (PCA) plot of transcriptome samples across all groups. **(B)** Bar plot showing the number of differentially expressed genes (DEGs) for each pairwise comparison. **(C)** Heatmap depicting the expression patterns of all identified DEGs across samples. **(D)** Heatmap of KEGG pathway enrichment results for DEGs between consecutive time points; the color of each square represents the false discovery rate (FDR), with red indicating statistical significance (FDR < 0.05) and green indicating non-significance (FDR ≥ 0.05).

Principal component analysis (PCA) revealed tight clustering of samples within each treatment group, indicating good reproducibility among biological replicates (**Figure 2A**). Differential expression analysis revealed that the downregulated genes consistently exceeded the upregulated genes in most pairwise comparisons (**Figures 2B, C**). The comparison between GA_6d and CK_0d yielded the highest number of differentially expressed genes (DEGs; 34,541), whereas the comparison between GA_12d and GA_6d identified the lowest number of DEGs (1,417) (**Figure 2B**).

To elucidate the dynamic transcriptional changes during internode development, we focused on the KEGG enrichment analysis of DEGs between consecutive time points (**Figure 2D**). The plant hormone signal transduction pathway was significantly enriched in multiple groups, including CK_6d vs. CK_0d, CK_12d vs. CK_6d, and GA_6d vs. CK_0d (**Figure 2D**). Concurrently, the brassinosteroid and phenylpropanoid biosynthesis pathways were significantly enriched in all comparisons (**Figure 2D**). The cutin, suberine, and wax biosynthesis pathways were significantly enriched in the CK_6d vs. CK_0d, CK_12d vs. CK_6d, and GA_12d vs. GA_6d comparisons (**Figure 2D**). Regarding primary metabolism, the starch and sucrose metabolic pathways were significantly enriched in the CK_6d vs. CK_0d, CK_12d vs. CK_6d, and GA_6d vs. CK_0d comparisons (**Figure 2D**). MAPK signaling pathway and alpha-linolenic acid metabolism were also significantly enriched in the CK_6d vs. CK_0d and GA_6d vs. CK_0d comparisons (**Figure 2D**).

### 3.3 Proteomic changes associated with internode development

**Figure 3:**
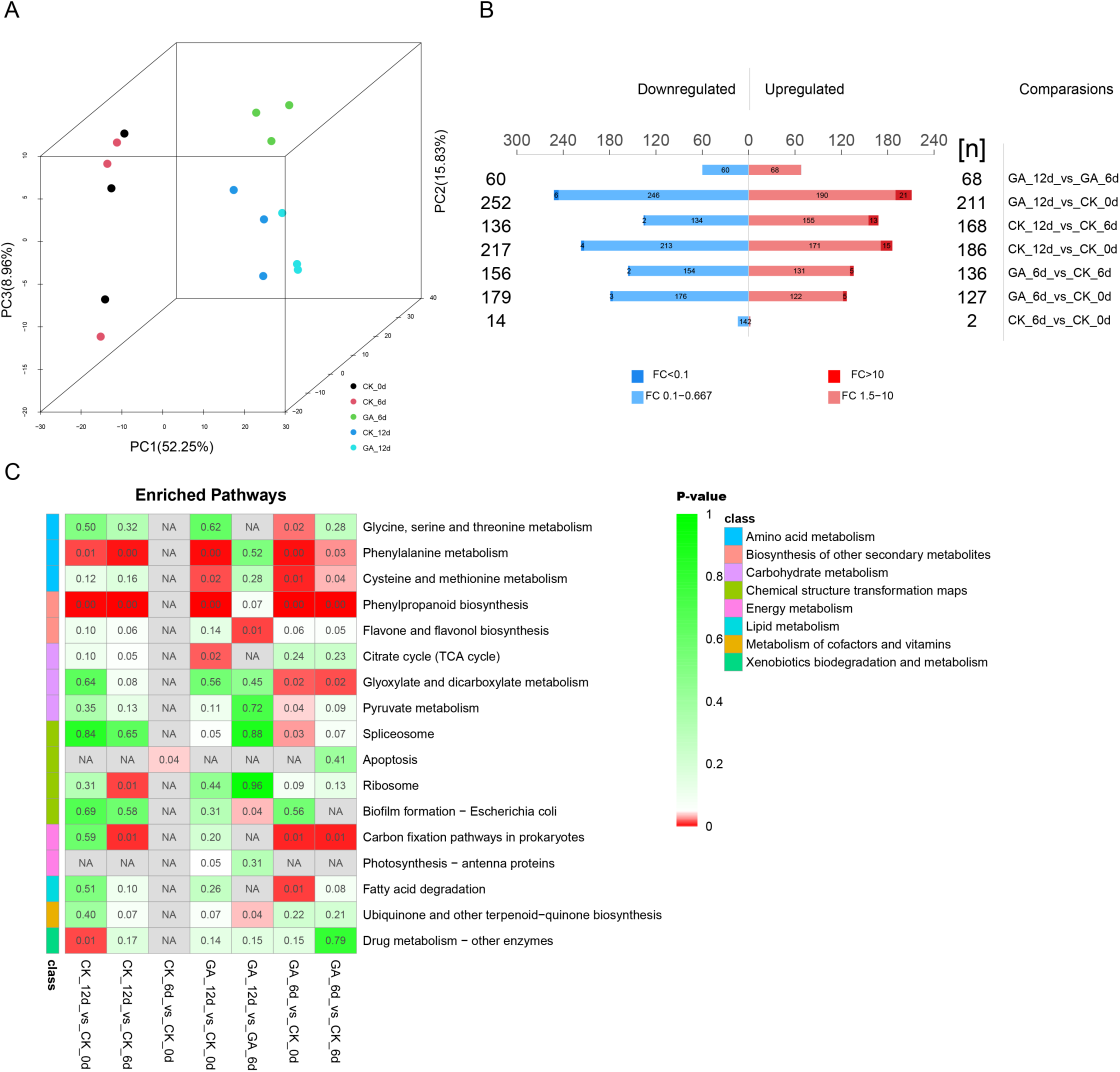
Proteomic profiling of sugarcane internodes during development and GA_3_ response. **(A)** 3D principal component analysis (PCA) plot of proteome samples across all groups. **(B)** Bar plot showing the number of differentially expressed proteins (DEPs) for each pairwise comparison. **(C)** Heatmap of KEGG pathway enrichment results for DEPs from each comparison, with red indicating statistical significance (FDR < 0.05) and green indicating non-significance (FDR ≥ 0.05).

A total of 1,430 proteins were identified by proteomic analysis (**Supplementary Table 2**). Principal component analysis (PCA) revealed a clear separation among most sample groups, except for CK_6d and CK_0d, which clustered closely together (**Figure 3A**). In most pairwise comparisons, most of the DEPs were downregulated (**Figure 3B**). The comparison between GA_6d and CK_0d yielded the highest number of differentially expressed proteins (363), whereas a relatively lower number of DEPs (128) was observed between GA_12d and GA_6d (**Figure 3B**). This pattern was consistent with the differential transcriptomic expression analysis (**Figure 2**).

KEGG enrichment analysis of the DEPs indicated that specific metabolic pathways were significantly enriched during internode development and in response to GA_3_ treatment (**Figure 3C**). The phenylpropanoid biosynthesis and phenylalanine metabolic pathways were the most prominent, showing significant enrichment during natural development (CK_12d vs. CK_0d, CK_12d vs. CK_6d) and across all major GA_3_ treatment comparisons (GA_6d vs. CK_6d, GA_6d vs. CK_0d, GA_12d vs. CK_0d) (**Figure 3C**). Notably, pathways linked to primary carbon and nitrogen metabolism, including carbon fixation pathways in prokaryotes, glyoxylate and dicarboxylate metabolism, and glycine, serine, and threonine metabolism, were enriched at the early stage (GA_6d vs. CK_0d) (**Figure 3C**). By the later stage of GA_3_ treatment (GA_12d vs. CK_0d), the citrate cycle (TCA cycle) was significantly enriched (**Figure 3C**). Furthermore, flavone and flavonol biosynthesis were specifically enriched in the “within-GA-treatment comparison” (GA_12d vs. GA_6d) (**Figure 3C**).

### 3.4 Integrative analysis of transcriptomic and proteomic data

**Figure 4:**
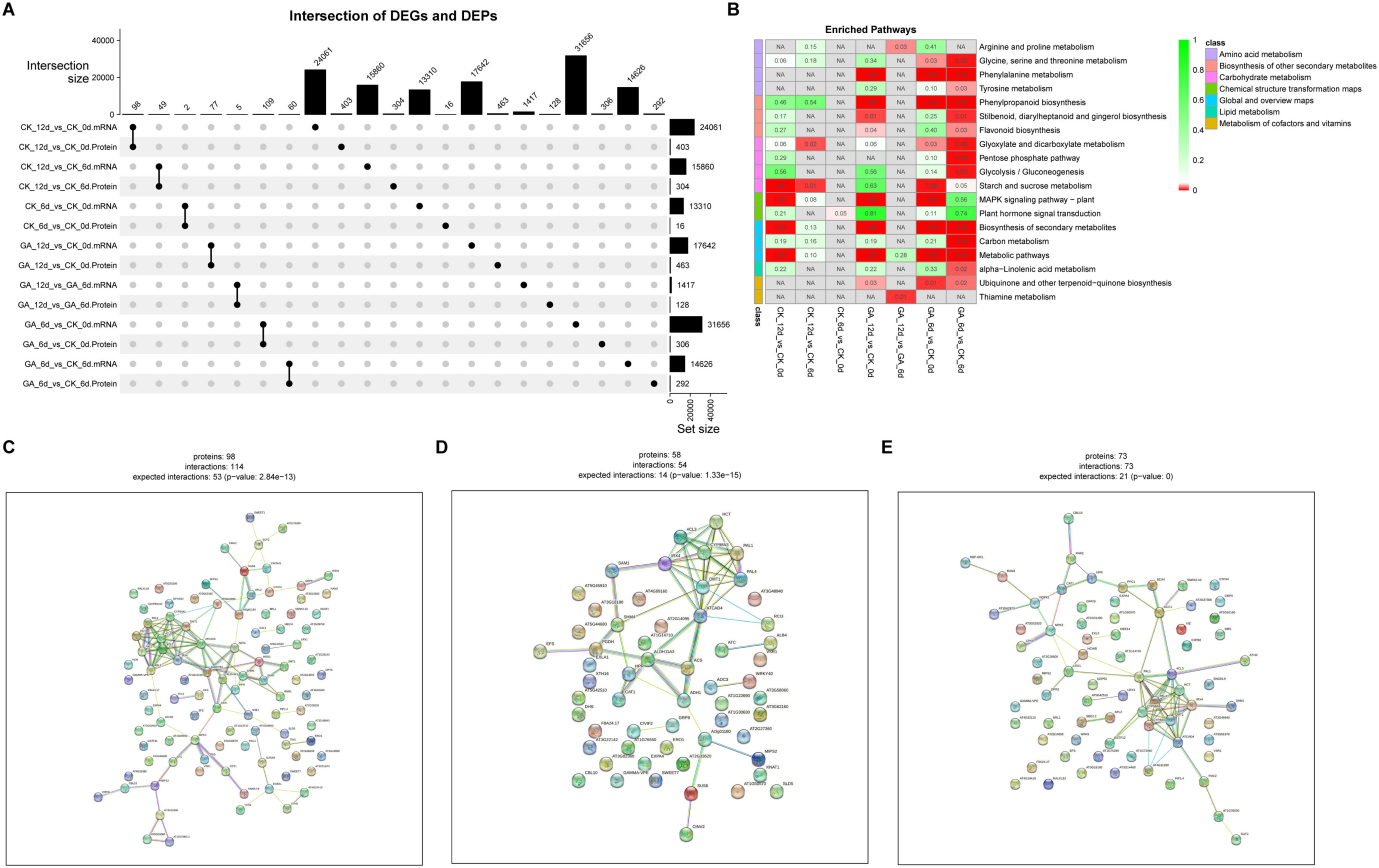
Integrative analysis of transcriptomic and proteomic data. **(A)** Intersection of differentially expressed genes (DEGs) and differentially expressed proteins (DEPs) for each pairwise comparison (UpSet plot). **(B)** Heatmap of KEGG pathway enrichment results for the intersecting gene sets from selected comparisons, with red indicating statistical significance (FDR < 0.05) and green indicating non-significance (FDR ≥ 0.05). **(C)** Protein-protein interaction (PPI) network of the intersecting genes/proteins in the GA_6d vs. CK_0d comparison. Nodes represent proteins (based on homology to *Arabidopsis*), and edges indicate predicted functional interactions. **(D)** PPI network of the intersecting genes/proteins in the GA_6d vs. CK_6d comparison. **(E)** PPI network of the intersecting genes/proteins in the GA_12d vs. CK_0d comparison.

Intersection of DEGs and DEPs from each pairwise comparison was performed (**Figure 4A**), followed by KEGG enrichment analysis (**Figure 4B**). The GA_6d vs. CK_6d overlap was enriched in phenylalanine metabolism, phenylpropanoid biosynthesis, flavonoid biosynthesis, glycolysis/gluconeogenesis, and glyoxylate and dicarboxylate metabolism (**Figure 4B**). The GA_6d vs. CK_0d overlap was enriched in phenylalanine metabolism, phenylpropanoid biosynthesis, starch and sucrose metabolism, and MAPK signaling pathway (**Figure 4B**). The GA_12d vs. CK_0d overlap also showed enrichment of phenylalanine metabolism, phenylpropanoid biosynthesis, and MAPK signaling pathway (**Figure 4B**).

To explore functional interactions among the proteins encoded by the intersecting DEGs and DEPs, we constructed PPI networks based on homology to *Arabidopsis* (**Figures 4C–E**). The networks for GA_6d vs. CK_0d, GA_6d vs. CK_6d and GA_12d vs. CK_0d were shown as they are the most representative for the early GA_3_ response (**Figures 4C–E**). In the GA_6d vs. CK_0d network, hub proteins included PAL1, PAL2, PAL4, 4CL3, 4CL5, and CYP84A1 (**Figure 4C**). Sequence alignment indicated that the *Arabidopsis* PAL proteins matched sugarcane sequences annotated as PAL; 4CL3 and 4CL5 corresponded to sugarcane 4CL; and CYP84A1 corresponded to sugarcane ferulate 5-hydroxylase (F5H). In the GA_6d vs. CK_6d network (**Figure 4D**) and the GA_12d vs. CK_0d network (**Figure 4E**), hub proteins included PAL1, PAL4, 4CL3, and ATCAD4. Their sugarcane counterparts were PAL, 4CL, and cinnamyl-alcohol dehydrogenase (CAD) (**Figures 4D, E**).

### 3.5 STEM trend analysis reveals divergent dynamic patterns in response to GA_3_ treatment

**Figure 5:**
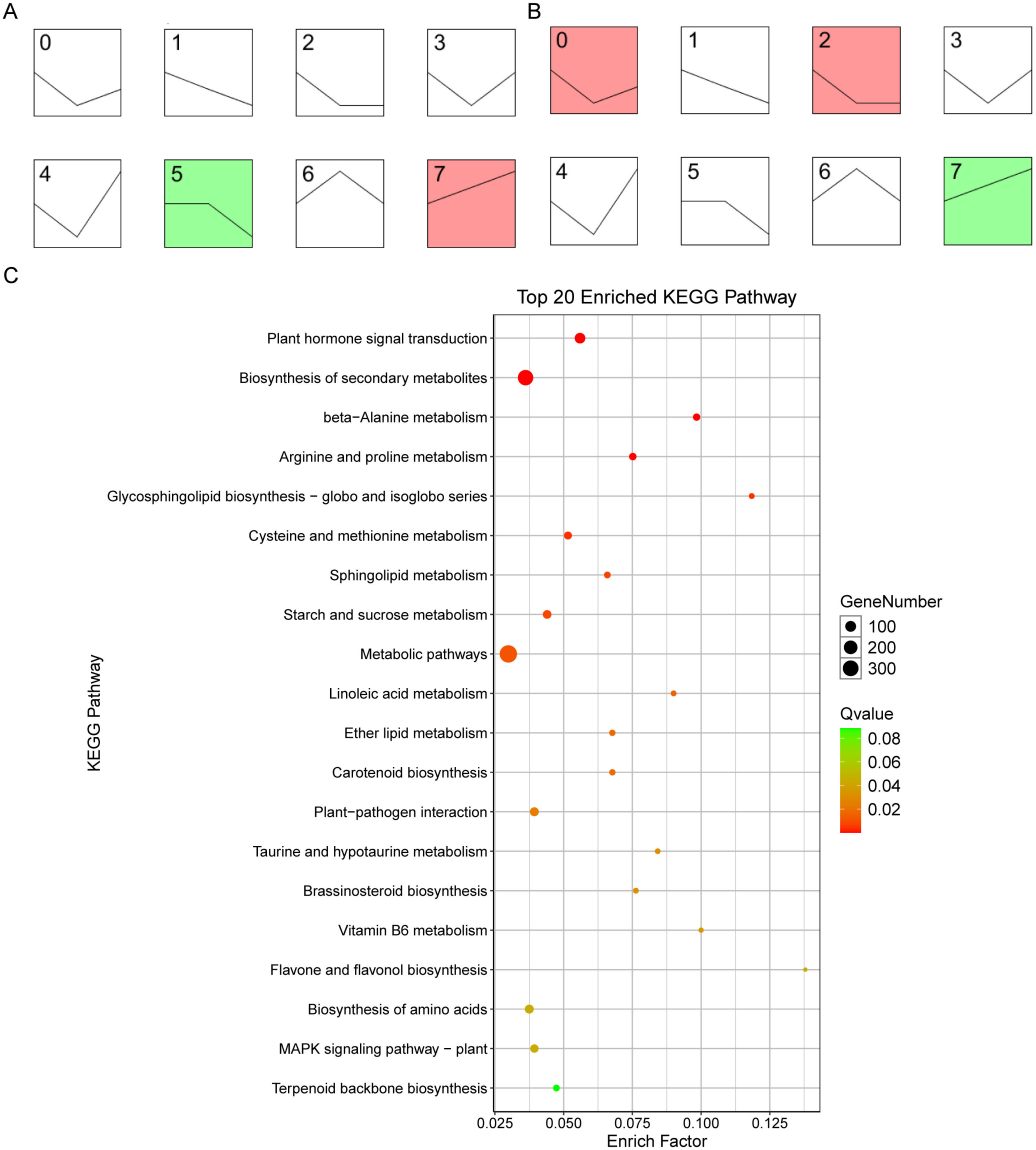
STEM trend analysis of transcriptomic dynamics. **(A)** Expression trend profiles (profiles 0-7) for genes in the control (CK) group over development time (0, 6, 12 days). **(B)** Expression trend profiles for genes in the GA_3_-treated (GA) group over time. **(C)** KEGG pathway enrichment analysis of genes exhibiting divergent expression trends between the CK and GA groups. In panels A and B, each colored block represents a statistically significant trend profile (Fisher’s exact test, *p* < 0.05), with profiles sharing the same color being merged into a single cluster (e.g., down_regain, down, up_regain, up). Grey blocks indicate non-significant profiles (*p* ≥ 0.05). The categorization of profiles into combined clusters (down_regain, down, up_regain, up, other) is as described in the Results.

**Figure 6:**
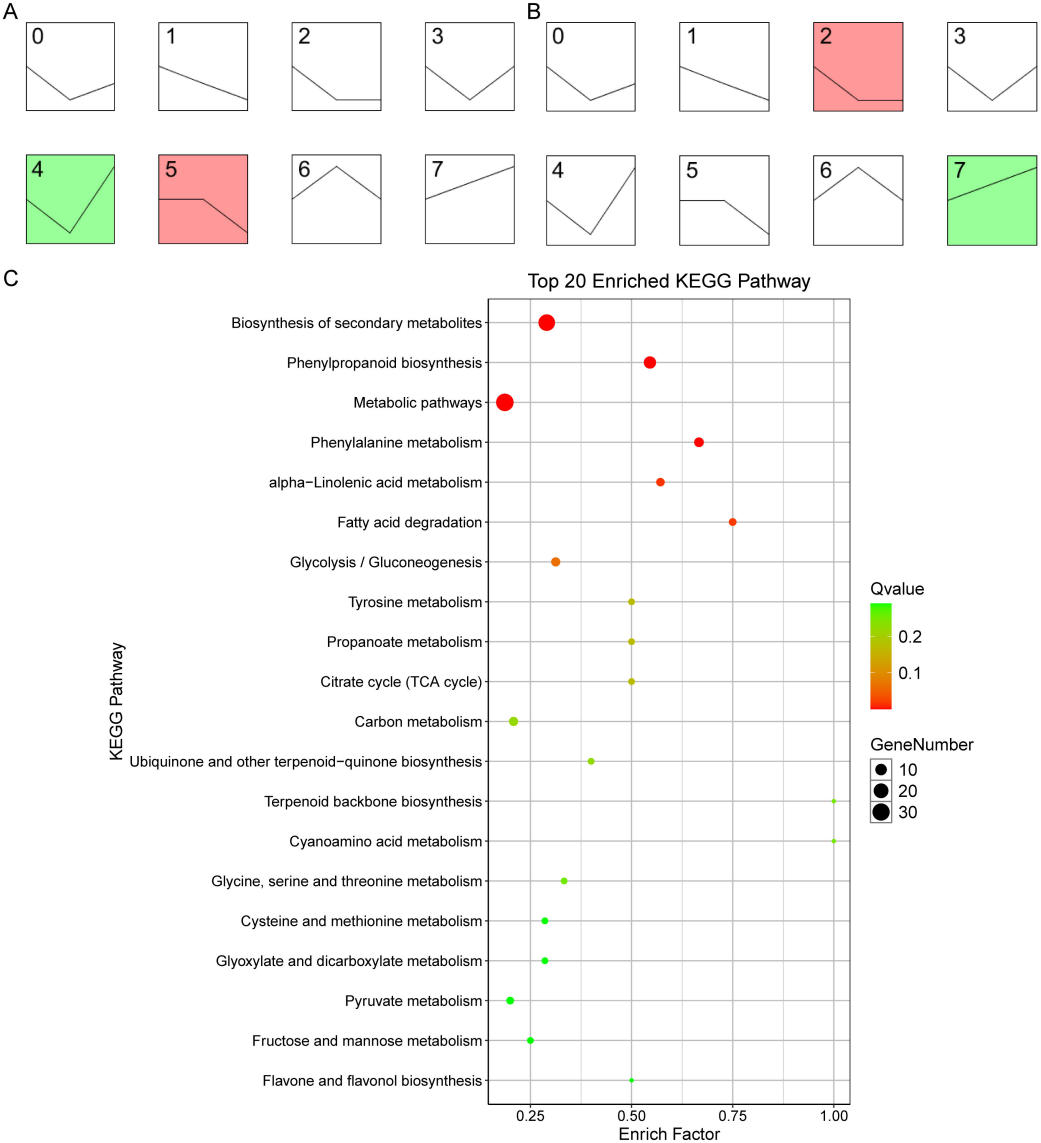
STEM trend analysis of proteomic dynamics. **(A)** Abundance trend profiles for proteins in the control (CK) group over time (0, 6, 12 days). **(B)** Abundance trend profiles for proteins in the GA_3_-treated (GA) group over time. **(C)** KEGG pathway enrichment analysis of proteins exhibiting divergent abundance trends between the CK and GA groups. In panels A and B, each colored block represents a statistically significant trend profile (Fisher’s exact test, *p* < 0.05), with profiles sharing the same color being merged into a single cluster (e.g., down_regain, down, up_regain, up). Grey blocks indicate non-significant profiles (*p* ≥ 0.05). The categorization of profiles into combined clusters (down_regain, down, up_regain, up, other) is as described in the Results.

The temporal expression trajectories of both transcripts and proteins were analyzed across the time series within the CK (CK_0d, CK_6d, CK_12d) and the GA_3_ treatment (GA) groups (CK_0d, GA_6d, GA_12d) (**Figures 5** and **6**). Gene expression profiles exhibited broadly similar trends between the CK and GA groups (**Figures 5A, B**). The profiles were categorized as follows: down_regain (profiles 0, 3, 4), down (profiles 1, 2, 5), up_regain (profile 6), and up (profile 7), with the remaining DEGs classified as ‘other.’ This analysis identified 4,597 genes displaying divergent expression trends between the CK and GA groups (**Supplementary Table 3**). KEGG enrichment analysis of these genes revealed the significant involvement of several key pathways, including plant hormone signal transduction, brassinosteroid biosynthesis, phenylpropanoid biosynthesis, and cutin, suberine, and wax biosynthesis (**Figure 5C**). Furthermore, pathways associated with central metabolism and signaling, such as starch and sucrose metabolism, the MAPK signaling pathway (plant), terpenoid backbone biosynthesis, and carotenoid biosynthesis, were also prominently enriched (**Figure 5C**).

A parallel trend analysis was performed at the protein level (**Figures 6A, B**). Using the same categorization method, 60 proteins with divergent abundance trends were identified between the CK and GA_3_ treatment groups (**Supplementary Table 4**). KEGG enrichment analysis of these proteins revealed significant enrichment for pathways, including phenylpropanoid biosynthesis, the citrate cycle (TCA cycle), glycolysis/gluconeogenesis, alpha-linolenic acid metabolism, and fatty acid degradation (**Figure 6C**).

### 3.6 Identification of core regulatory genes and proteins in GA_3_ response

**Figure 7:**
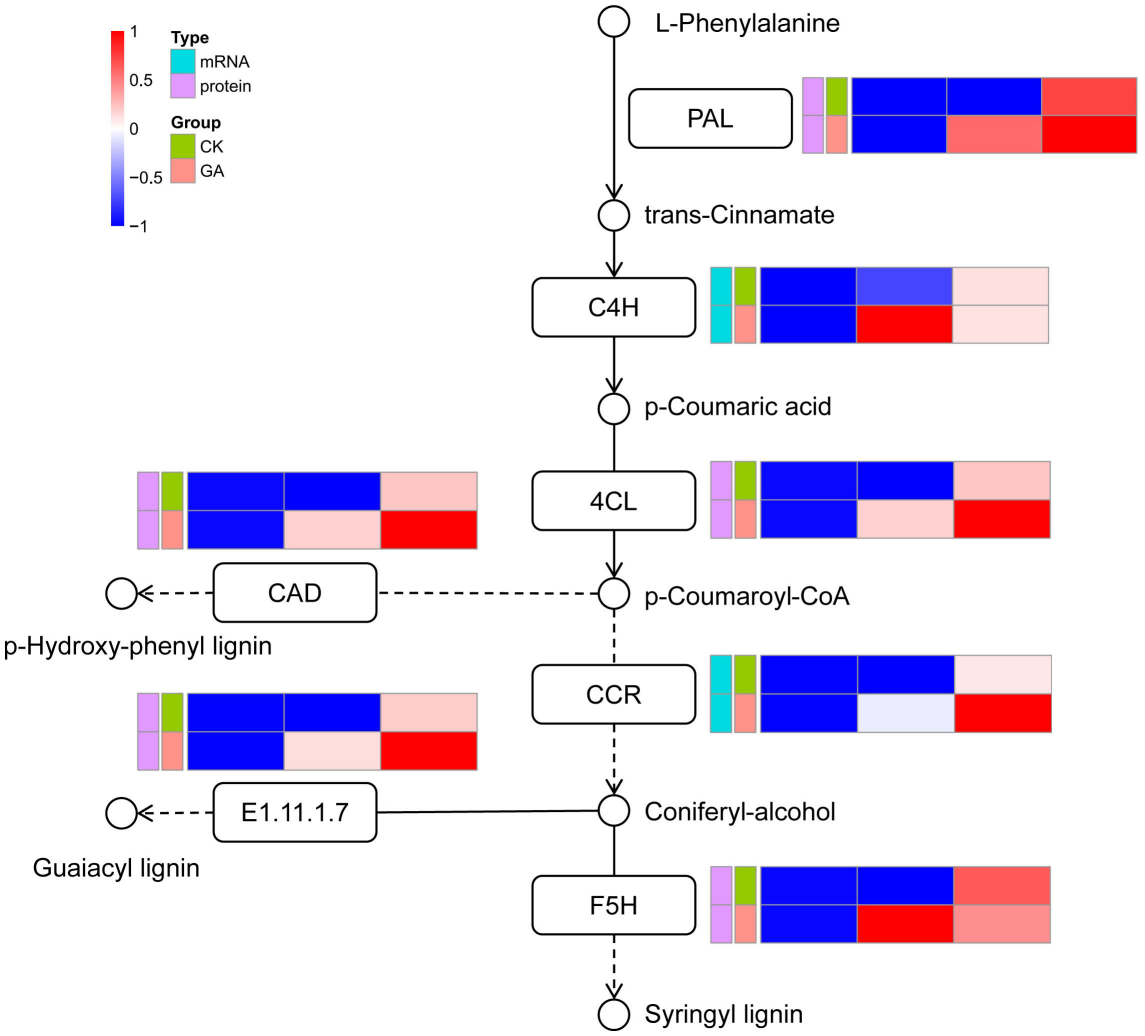
Dynamic reprogramming of the phenylpropanoid biosynthesis pathway in response to GA_3_. The network illustrates the core metabolic route from L-phenylalanine to the synthesis of the major monolignol intermediates leading to lignin polymerization. Integrated heatmaps adjacent to each gene represent its corresponding mRNA or protein abundance levels for the control (CK, upper row) and the GA_3_-treated (GA, lower row) groups over time (0d, 6d, and 12d) from left to right. The color gradient from blue to red indicates low to high expression/abundance, respectively.

Given the consistent and significant enrichment of the phenylpropanoid biosynthesis pathway across our analyses of DEGs, DEPs, STEM trends, and intersecting genes/proteins with divergent trends between the CK and GA_3_ treatment groups (**Figures 2**-**6**), we constructed a detailed metabolic network to delineate the molecular basis of GA_3_-induced internode maturation (**Figure 7**). The schematic traces the metabolic flow from L-phenylalanine to the synthesis of three major lignin monomers: p-hydroxyphenyl (H), guaiacyl (G), and syringyl (S) lignin (**Figure 7**). The expression level or protein abundance of *PAL*, *4CL*, *CCR*, *CAD*, and the peroxidase (E1.11.1.7) genes, exhibited a time-dependent increase in both CK and GA_3_-treated groups (**Figure 7**). Notably, this upregulation was markedly accelerated by GA_3_ treatment, with significantly higher levels evident at 6 d post-treatment (**Figure 7**). In contrast, the expression dynamics of *C4H* and *F5H* peaked in GA_6d and subsequently declined in GA_12d group, whereas in the CK group, their accumulation was delayed in CK_12d (**Figure 7**). These results indicate that exogenous GA_3_ not only enhances the overall flux through the phenylpropanoid pathway but also differentially modulates the temporal expression of specific branch-point enzymes, potentially steering the metabolic output during rapid internode elongation.

**Figure 8:**
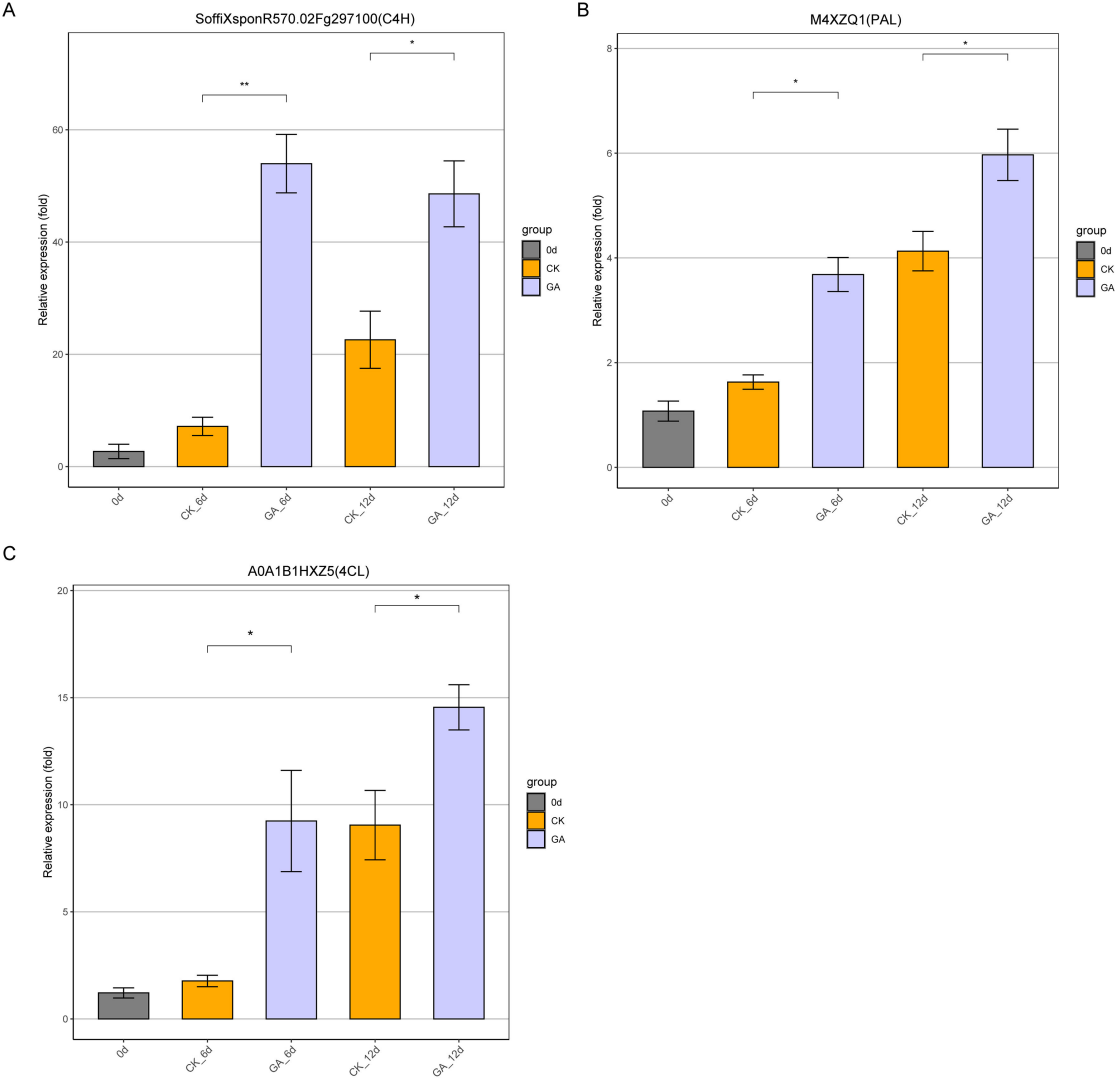
Validation of key phenylpropanoid biosynthesis gene expression by RT-qPCR. Relative mRNA expression levels of trans-cinnamate 4-monooxygenase (*C4H*) **(A)**, phenylalanine ammonia-lyase (*PAL*) **(B)**, and 4-coumarate-CoA ligase 1 (*4CL*) **(C)** and 4-coumarate-CoA ligase 1 (*4CL*) in internode samples from control (CK) and GA_3_-treated (GA) groups at 0, 6, and 12 days post-treatment. Data are presented as mean ± SD (n = 3 biological replicates). Asterisks denote significant differences between the GA_3_-treated and control samples at the same time point as determined by Student’s t-test (*p < 0.05, **p < 0.01).

To validate the findings of our transcriptomic and proteomic analyses, we performed RT-qPCR assays on three core genes involved in the phenylpropanoid biosynthesis pathway: phenylalanine ammonia-lyase (*PAL*), trans-cinnamate 4-monooxygenase (*C4H*), and 4-coumarate-CoA ligase 1 (*4CL*). The results confirmed that the expression dynamics of these genes were consistent with the multi-omics trends (**Figure 8**).

### 3.7 Biochemical and mechanical validation of GA_3_-induced internode maturation

**Figure 9:**
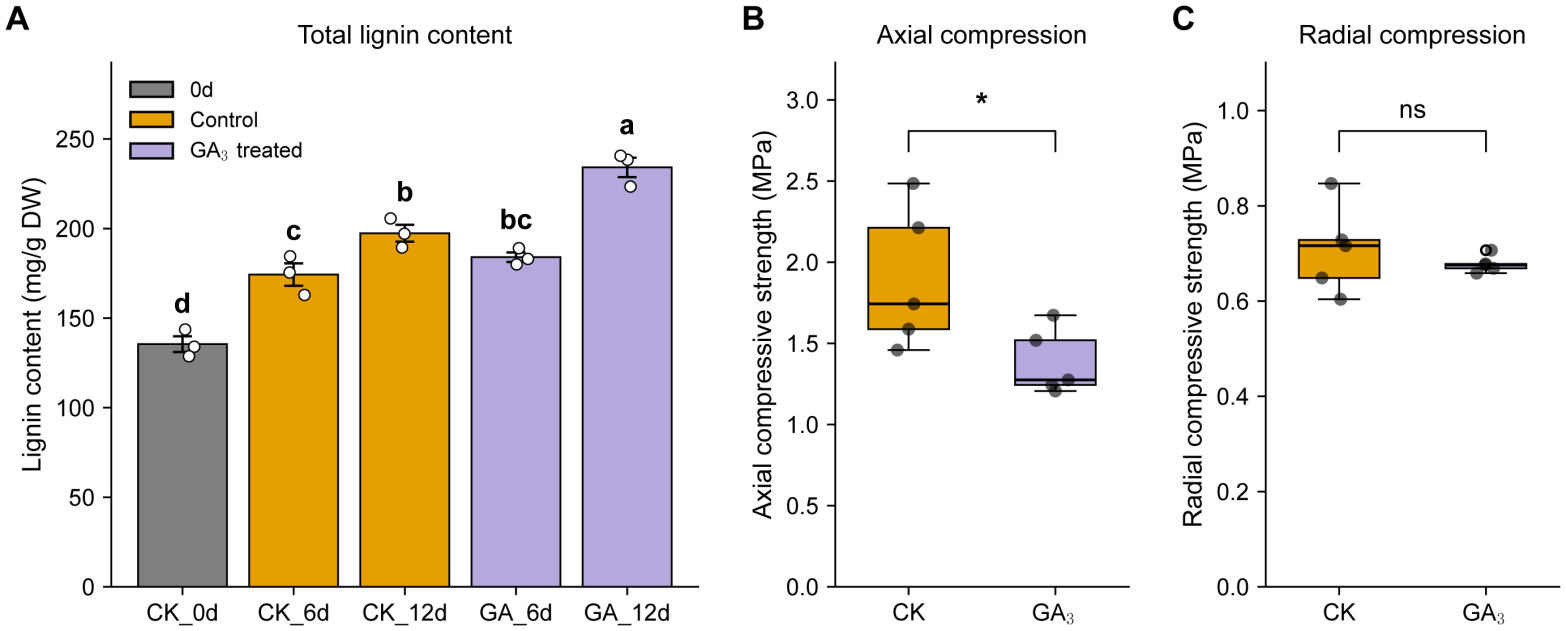
Biochemical and mechanical validation of GA_3_-induced internode maturation. **(A)** Total lignin content (mg/g dry weight) of internode tissues from CK_0d, CK_6d, GA_6d, CK_12d, and GA_12d groups, measured by the acetyl bromide method. Data are presented as bar plots with error bars representing standard deviation (n = 3 biological replicates). Different lowercase letters above the bars indicate significant differences among the five groups (one-way ANOVA followed by Tukey’s HSD *post-hoc* test, *p* < 0.05); the letter “a” denotes the highest mean. **(B, C)** Boxplots showing **(B)** axial and **(C)** radial compressive strength (MPa) of internode segments from control (CK) and GA3-treated (GA) groups at 6 d post-treatment (n = 5 biological replicates). For each boxplot, the box represents the interquartile range (IQR), the horizontal line indicates the median, and the whiskers extend to the minimum and maximum values. Asterisks denote significant differences between control and GA_3_-treated groups as determined by two-tailed Student’s t-test (*p < 0.05; ns, not significant).

To biochemically corroborate the omics-predicted activation of phenylpropanoid biosynthesis, total lignin content in internode tissues was determined using the acetyl bromide method (**Figure 9A**). CK_0d showed the lowest lignin content, with progressive increases from CK_0d to CK_6d to CK_12d (Tukey’s HSD, *p* < 0.05). GA_6d was significantly higher than CK_0d but comparable to CK_6d and CK_12d, whereas GA_12d exhibited the highest lignin content. These data are consistent with the up-regulation of PAL, 4CL, and CCR observed at both the transcript and protein levels, supporting accelerated lignin deposition during GA_3_-induced internode maturation.

To assess whether the early biochemical changes translated into mechanical phenotypes, axial and radial compression tests were performed at 6 d post-treatment. Axial compressive strength (MPa) was significantly lower in the GA_3_-treated plants than in controls, while radial compressive strength was unchanged (**Figures 9B, C**; Student’s t-test; axial *p* = 0.044, radial *p* = 0.480), suggesting that GA_3_-induced acceleration of lignin biosynthesis had not yet translated into a measurable change in stem mechanical strength during the rapid elongation phase examined here.

## 4 Discussion

The implementation of multi-faceted strategies based on GA_3_ induction is widely used in agricultural and horticultural practices to improve crop yield and product quality. However, GA_3_ application and evaluation for specific crop species rely on comprehensive biological investigations. According to our findings, GA_3_ primarily encourages internodal elongation rather than stalk diameter expansion during the peak elongation stage. The growth-promoting effect of GA_3_ on the internode elongation of sugarcane has been reported previously (Verma et al., 2017; Qiu et al., 2019; Chen et al., 2020). In this study, we integrated transcriptomic and proteomic datasets to comprehensively investigate the changes in gene expression and protein abundance during GA_3_-induced internode development in sugarcane and systematically elucidated the underlying multi-omics regulatory mechanism.

To characterize the global transcriptional patterns across experimental conditions, we conducted PCA on the full set of gene expression profiles from all groups. This analysis revealed a clear separation of the CK_0d, CK_6d, and CK_12d from GA_6d and GA_12d, indicating significant transcriptional differentiation between CK and treatment groups. In addition, a large number of DEGs were detected in GA_6d vs. CK_0d, and GA_12d vs. CK_0d. Thus, from the gene expression perspective, GA_3_ was found to have a significant regulatory effect on the transcriptional activity of genes associated with internode development. Conversely, the GA_6d and GA_12d samples clustered together, with only a small number of DEGs detected in this comparison, indicating that the GA_3_-induced transcriptional changes in sugarcane had stabilized by 6 d post-treatment.

Building on the global expression-level changes described above, we next examined the underlying biological pathways through enrichment analysis and identified the pathways associated with the DEGs. GA_3_ stimulated sugarcane internode elongation by regulating the genes associated with phenylpropanoid biosynthesis, which is responsible for monolignol synthesis and lignin polymer deposition, and further reinforced the mechanical rigidity of plant cell walls and contributed to the maturation of sugarcane stems. *PAL* gene (AGF69114) transcript level showed significant differences in GA_6d vs. CK_6d and GA_12d vs. CK_6d. These results were validated by performing RT-qPCR. Using RNA interference (RNAi), Cass et al. generated BdPAL-silenced plants, in which lignin content was reduced by 43% and cell wall−bound ferulate content by 57% in stem cell walls (Cass et al., 2015). The *CCR* gene (CAA13176) was up-regulated in GA_6d compared to that in CK_0d and CK_6d. It has also been reported that knockout mutant lines of *Arabidopsis thaliana* targeting this gene display a dwarf phenotype, accompanied by a 25-35% decrease in total lignin content (Jones et al., 2001; Mir Derikvand et al., 2008). The levels of these two DEGs were preliminarily consistent with the changing trend of the tested lignin content. When compared with CK_0d, the major representation of genes in CK_6d and GA_6d was found to be under the plant hormone signal transduction pathway, indicating the function of these genes in the regulation of plant height. This process involves a coordinated regulation mediated by interactions with other endogenous plant hormones (Wu et al., 2009; Qiu et al., 2019). The cutin, suberin, and wax biosynthesis pathway also act as a critical regulatory pathway that mediates GA_3_-induced internode development. These results were consistent with those of a previous study (Chen et al., 2020). Related water-repellent polymers, cutin and suberin are made of long-chain hydroxy or epoxy fatty acids (C16, C18) that are cross-linked by ester groups. Cutin, the core skeletal component of the cuticle, is localized in the primary cell walls of sugarcane stems and exerts structural support. Waxes, a complex mixture of very long-chain lipids with carbon chain lengths ranging from C25 to C35, form a wax cuticle that coats the outer surface of the plant cuticle. Importantly, they exhibit strong hydrophobicity and act as an essential hydrophobic barrier that enhances plant resistance to various abiotic stresses.

To independently confirm that the transcriptional reprogramming was reflected at the protein level, the proteomic analysis was concurrently conducted, and the phenylpropanoid biosynthesis was the most significantly upregulated pathway induced by GA_3_. As crucial enzymes, PAL (A0A9E8ADM7, M1MQ13, M4XZQ1, U3M000, W5RSK7, and W5RSQ2) was upregulated in GA_3_ treatment group compared to the CK group. Silva et al. demonstrated that reduced expression of PAL in the sugarcane roots may compromise the structural integrity of root cells through impairing lignin biosynthesis, thereby rendering them more susceptible to oxidative stress (Silva et al., 2025). Besides, the expression of CCR (O82055) was significantly increased (GA_6d vs. CK_0d, GA_6d vs. CK_6d, GA_12d vs CK_0d, and GA_12d vs. GA_6d). Tobacco lines with decreased steady-state levels of CCR mRNA and attenuated CCR activity exhibited a significant reduction in lignin content, accompanied by a range of developmental abnormalities including reduced stem size, aberrant leaf morphology, and vessel collapse (Piquemal et al., 1998). The levels of these two DEPs were preliminarily consistent with the changing trend of the tested lignin content. Although other pathways are intrinsically necessary for the regular growth of sugarcane, phenylalanine metabolism acts upstream of phenylpropanoid biosynthesis, supplies it with its exclusive starting substrate (L-phenylalanine), and has a more noticeable stimulatory influence on internode development under GA_3_ control. Finally, the expression of 3-O-methyltransferase (Q6UNM7 and O82054), which is involved in flavone and flavonol biosynthesis, was increased (GA_6d vs. CK_0d, GA_6d vs. CK_6d, GA_12d vs. CK_0d, and GA_12d vs. GA_6d). Flavone and flavonol biosynthesis represent a key downstream branch derived from the phenylpropanoid pathway, which shares the common upstream precursors and is initiated by specific enzymes committed to flavonoid production. Flavonoids and lignin compete for common metabolic flux, and flavonoid-lignin homeostasis acts as a key regulator of the growth-defense trade-off in plants (Yang et al., 2025).

Convergent evidence from the intersection of DEGs and DEPs, the STEM time-series trend analysis, and the PPI homology network further showed the phenylpropanoid biosynthesis pathway under GA_3_ induction. It is essential for the synthesis of lignin and the development of sugarcane internodes. Additionally, PPI analysis revealed high-confidence interactions between two hub proteins (PAL and 4CL). These interactions are supported by KEGG database. L-phenylalanine is converted to trans-cinnamate by PAL, followed by hydroxylation to p-coumaric acid via C4H, and subsequent activation to p-coumaroyl-CoA by 4CL. In addition to differences at the protein expression level, qPCR analysis verified that the transcript abundance of the *4CL* gene was significantly different between the GA_3_-induced and control groups. Multiple studies have demonstrated that 4CL serves as a key branch-point enzymes in phenylpropanoid biosynthesis and that the biosynthesis of lignin and flavonoids shares this key intermediate (Hamberger and Hahlbrock, 2004; Meng et al., 2024). Finally, our multi-omics data suggested that p-coumaroyl-CoA is converted into p-hydroxyphenyl lignin catalyzed by CAD. For the biosynthesis of guaiacyl lignin and syringyl lignin, p-coumaroyl-CoA is first transformed into coniferyl alcohol under the catalysis of CCR, and further generates the corresponding lignin polymers via E1.11.1.7 and F5H, respectively. There was a highly significant correlation between the two genes with upregulated transcript levels and five proteins with increased expression identified in this pathway, and the elucidation and analysis of this regulatory pathway are of great significance for exploring the molecular mechanisms underlying sugarcane internode development and maturation.

The temporal pattern of the GA_3_ response, in which a marked transcriptomic and proteomic divergence at 6 d stabilizes by 12 d and is accompanied by an accelerated lignin trajectory that exceeds natural development at the later time point, can be interpreted in the context of the developmental progression of the +2 internode. Sugarcane internode elongation proceeds through a sequence of phases in which the actively elongating internode is associated with elevated endogenous gibberellin and reduced abscisic acid and ethylene levels, and this hormonal balance progressively shifts as the internode enters secondary cell-wall deposition (Wu et al., 2009; Qiu et al., 2019). The 6-d sampling point coincides with the peak of this elongation phase, during which exogenous GA_3_ acts in synergy with the high endogenous GA pool to maximally upregulate the phenylpropanoid pathway at both the transcript and protein level (PAL, C4H, 4CL, CCR, CAD; **Figures 2D**, **3C**, and **7**); the corresponding total-lignin content in the +2 internode at 6 d reaches a level comparable to that of untreated controls at 12 d (**Figure 9A**), effectively compressing approximately six days of natural maturation into the early treatment window. By 12 d the omics differential expression has stabilized, with only 1,417 DEGs and 128 DEPs distinguishing GA_12d from GA_6d, far fewer than the 34,541 DEGs and 363 DEPs of the early GA response (**Figures 2B**, **3B**); this indicates that the +2 internode is approaching a developmental endpoint for further molecular-level GA_3_ modulation, while the downstream lignin polymer continues to accumulate and yields a statistically significant increase over controls at 12 d (**Figure 9A**). The same developmental gradient is reflected, at a coarser spatial scale, in the well-documented compositional change along the sugarcane stalk from immature apical to fully lignified basal internodes (Lingle and Thomson, 2012; Pereira et al., 2017), indicating that the timing and magnitude of GA_3_-induced lignification are governed by an interaction between the exogenous GA pulse and the endogenous developmental state of the target internode (including its position along the stalk, plant age, and the local hormone milieu) rather than by GA_3_ alone. This is also consistent with the broader cross-regulation between the phenylpropanoid and flavonoid branches that has been proposed to integrate growth and cell-wall maturation programs (Yang et al., 2025).

Several limitations of this study should be acknowledged. First, the time window is short: sampling covered only 0, 6, and 12 days after GA_3_ application, capturing the rapid-response and early-plateau phases of internode elongation; longer-term outcomes (specifically, the trajectory of lignification and sucrose accumulation across the full maturation period of 20–30 days and into the ripening stage) therefore cannot be evaluated from the present data. Second, the spatial sampling is restricted to the +2 internode and does not extrapolate to mature basal internodes whose properties are governed by later regulatory programs. Third, total lignin content was quantified without resolving H-, G-, and S-monomer composition, and lignin compositional remodeling therefore remains to be characterized in follow-up work. Fourth, direct functional validation of the core regulatory genes (PAL, C4H, 4CL, CCR, CAD), through approaches such as gene knockout, overexpression, or *in-vivo* enzyme assays, was beyond the scope of the present study and should be addressed in future work.

Notwithstanding these limitations, our integrated transcriptomic and proteomic analysis revealed the molecular mechanism by which exogenous GA_3_ regulates sugarcane internode maturation through the phenylpropanoid biosynthesis and phenylalanine metabolic pathways, with 4CL and PAL as the hub components. These findings not only deepen our understanding of GA_3_-mediated secondary metabolism regulation in sugarcane, but also identify core regulatory factors through multi-omics integration that can act as molecular markers or gene-editing targets, thus accelerating sugarcane molecular breeding and shortening the cycle of conventional breeding, while providing fundamental insights for yield improvement and industrial upgrading in sugarcane agricultural production.

## Data Availability

The transcriptomic raw sequencing data generated for this study were deposited into the NCBI Read Archive under BioProject accession No. PRJNA1437277. The proteomic mass spectrometry data were available in the iProX - Integrated Proteome Resources under accession No. IPX0016161001.
